# Partial Order Optimum Likelihood (POOL): Maximum Likelihood Prediction of Protein Active Site Residues Using 3D Structure and Sequence Properties

**DOI:** 10.1371/journal.pcbi.1000266

**Published:** 2009-01-16

**Authors:** Wenxu Tong, Ying Wei, Leonel F. Murga, Mary Jo Ondrechen, Ronald J. Williams

**Affiliations:** 1 College of Computer and Information Science, Northeastern University, Boston, Massachusetts, United States of America; 2 Institute for Complex Scientific Software, Northeastern University, Boston, Massachusetts, United States of America; 3 Department of Chemistry and Chemical Biology, Northeastern University, Boston, Massachusetts, United States of America; Stanford University, United States of America

## Abstract

A new monotonicity-constrained maximum likelihood approach, called Partial Order Optimum Likelihood (POOL), is presented and applied to the problem of functional site prediction in protein 3D structures, an important current challenge in genomics. The input consists of electrostatic and geometric properties derived from the 3D structure of the query protein alone. Sequence-based conservation information, where available, may also be incorporated. Electrostatics features from THEMATICS are combined with multidimensional isotonic regression to form maximum likelihood estimates of probabilities that specific residues belong to an active site. This allows likelihood ranking of all ionizable residues in a given protein based on THEMATICS features. The corresponding ROC curves and statistical significance tests demonstrate that this method outperforms prior THEMATICS-based methods, which in turn have been shown previously to outperform other 3D-structure-based methods for identifying active site residues. Then it is shown that the addition of one simple geometric property, the size rank of the cleft in which a given residue is contained, yields improved performance. Extension of the method to include predictions of non-ionizable residues is achieved through the introduction of environment variables. This extension results in even better performance than THEMATICS alone and constitutes to date the best functional site predictor based on 3D structure only, achieving nearly the same level of performance as methods that use both 3D structure and sequence alignment data. Finally, the method also easily incorporates such sequence alignment data, and when this information is included, the resulting method is shown to outperform the best current methods using any combination of sequence alignments and 3D structures. Included is an analysis demonstrating that when THEMATICS features, cleft size rank, and alignment-based conservation scores are used individually or in combination THEMATICS features represent the single most important component of such classifiers.

## Author Summary

Genome sequencing has revealed the codes for thousands of previously unknown proteins for humans and for hundreds of other species. Many of these proteins are of unknown or unclear function. The information contained in the genome sequences holds tremendous potential benefit to humankind, including new approaches to the diagnosis and treatment of disease. In order to realize these benefits, a key step is to understand the functions of the proteins for which these genes hold the code. A first step in understanding the function of a protein is to identify the functional site, the local area on the surface of a protein where it affects its functional activity. This paper reports on a new computational methodology to predict protein functional sites from protein 3D structures. A new machine learning approach called Partial Order Optimum Likelihood (POOL) is introduced here. It is shown that POOL outperforms previous methods for the prediction of protein functional sites from 3D structures.

## Introduction

Development of function prediction capabilities is a major challenge in genomics. Structural genomics projects are determining the 3D structures of expressed proteins on a high throughput basis. However, the determination of function from 3D structure has proved to be a challenging task; the functions of most of these structural genomics proteins remain unknown. Computationally based predictive methods can help to guide and accelerate functional annotation. The first step toward the prediction of the function of a protein from its 3D structure is to determine its local site of interaction where catalysis and/or ligand recognition occurs. Such capabilities have many important practical implications for biology and medicine.

We have reported on THEMATICS [1]–[4], for Theoretical Microscopic Titration Curves, a technique for the prediction of local interaction sites in a protein from its three-dimensional structure alone. In the application of THEMATICS, one begins with the 3D structure of the query protein, solves the Poisson-Boltzmann (P-B) equations using well-established methods, then performs a hybrid procedure to compute the proton occupations of the ionizable sites as functions of the pH. Residues involved in catalysis and/or recognition have different chemical properties from ordinary residues. In particular, these functionally important residues have anomalous theoretical proton occupation curves. THEMATICS exploits this difference and utilizes information from the shapes of the theoretical titration curves of the ionizable residues, as calculated approximately from the computed electrical potential function.

THEMATICS utilizes only the 3D structure of the query protein as input; neither sequence alignments nor structural comparisons are used. Recently it was shown [4] that, among the methods based on the 3D structure of the query protein only, THEMATICS achieves by far the best performance, as measured by sensitivity and precision for annotated catalytic residues.

The purpose of the present paper is five-fold: (1) We present a monotonicity-constrained maximum likelihood approach, called Partial Order Optimum Likelihood (POOL), to improve performance and expand the capabilities of active site prediction. (2) Then it is shown that POOL, with THEMATICS input data alone, outperforms previous statistical [4] and Support Vector Machine (SVM) [5] implementations of THEMATICS when applied to a test set of annotated protein structures. (3) It is then demonstrated that the inclusion of one additional 3D-structure-based feature, representing the ordinal size of the surface cleft to which each residue belongs, can result in some improved performance, as demonstrated by ROC curves and validated by Wilcoxon signed-rank tests. (4) With the introduction of environment features, POOL then can use the THEMATICS data to predict both ionizable and non-ionizable residues. This all-residues extension of THEMATICS, together with a cleft size rank feature, results in a simple 3D-structure-based functional site predictor that performs better than other 3D structure based methods and nearly as well as the very best current methods that utilize both the 3D structure and sequence homology. (5) Finally, the POOL approach is able to take advantage of sequence alignment-based conservation scores, when available, in addition to these structure-based features. When this additional information is included, the resulting classifier is shown to outperform all other currently available methods using any combination of structure and sequence information.

### THEMATICS Features

In prior implementations of THEMATICS for the identification of active-site residues from the 3D structure of the query protein [3]–[5], titration curve shapes were described by the moments of their first derivative functions. These first derivative functions are essentially probability density functions and give unity when integrated over all space. In Ko *et al.* [3], the third and fourth central moments μ_3_ and μ_4_ of these probability functions were used. These moments measure asymmetry (skewness) and, roughly, the area under the tails relative to the area near the mean (kurtosis), respectively. In Tong *et al.* [5], the first moment and second central moment were also used. In each of these approaches, the moments measure deviations from normal curve shape and the analyses identify the outliers, the residues that deviate most from the normal proton occupation behavior. These prior approaches all use spatial clustering, so that outlier residues are reported as positive by the method if and only if they are in sufficiently close spatial proximity to at least one other outlier. Thus the previous THEMATICS identifications involve two stages, where the first stage makes a binary (outlier / not an outlier) decision on each residue and the second stage finds spatial clusters of the outliers. In the new approach reported here, every residue is assigned a probability that it is an active-site residue. Here, as an alternative to the clustering approach, we introduce features that describe a residue's neighbors; we call these *environment features*. For a given scalar feature x, we define the value of the environment feature x^env^(r) for a given residue r to be:
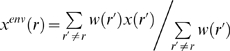
(1)where r′ is an ionizable residue whose distance d(r′,r) to residue r is less than 9Å, and the weight w(r′) is given by 1/d(r′,r)^2^.

In this study, we use the same features μ_3_ and μ_4_ used in the Ko [3] approach, along with the additional features μ_3_^env^ and μ_4_^env^. Thus every ionizable residue in any protein structure is assigned the 4-dimensional feature vector (μ_3_, μ_4_, μ_3_^env^, μ_4_^env^). The present approach has a number of advantages. Specifically, active residues may be selected in one step and they can be rank-ordered according to the probability of involvement in an active site. Furthermore, while THEMATICS previously has been applied to ionizable residues only, the present approach opens the door to direct prediction of non-ionizable active site residues, because the environment features μ_3_^env^ and μ_4_^env^ are well defined for all residues, including the non-ionizable ones. Finally, additional geometric features that are obtainable from the 3D structure only may be readily combined with the four THEMATICS features in order to enhance performance.

Geometric features, such as the relative sizes of the clefts on the surface of the protein structure, have been shown to correlate with active site location [6],[7]. For instance, for the majority of single-chain proteins, the catalytic residues are in the largest cleft. However geometric features alone do not perform comparatively well for active residue prediction, particularly because they are not very selective. It is shown here that cleft size information combined with THEMATICS electrostatic features yields high performance in purely 3D structure based functional site predictions.

### Monotonicity Assumptions for THEMATICS Features

The monotonicity-constrained maximum-likelihood approach underlying the POOL method described below is built on certain assumptions relating features used for classification to the probability that an instance having those features belongs to the positive class. Here we describe in detail the form these assumptions take when relating the THEMATICS features listed above to the probability that a residue is an active-site residue. Later we will also note that similar assumptions are reasonable when considering cleft rank and sequence conservation scores and apply them to those features as well. These THEMATICS feature-based monotonicity assumptions are as follows:

1. Given two ionizable residues in a single protein, the one having the more perturbed titration curve is more likely to be an active-site residue, all other things being equal.
2. Given two residues in a single protein, the one having a greater degree of overall titration curve perturbation among the ionizable residues in its spatial vicinity is more likely to be an active-site residue, all other things being equal.

More precisely, for the first assumption, we treat μ_3_ and μ_4_ as measures of degree of perturbation, and for the second we treat μ_3_^env^ and μ_4_^env^ as measures of overall perturbation within the spatial vicinity. These assumptions then become: Given two residues in the same protein, let their corresponding 4-dimensional feature vectors be **x** = (x_1_, x_2_, x_3_, x_4_) and **y** = (y_1_,y_2_, y_3_, y_4_). If x_i_≤y_i_ for each i, the probability that the first residue is an active-site residue is less than or equal to the probability that the second residue is an active-site residue. A more elegant formulation arises from the definition of a coordinate-wise partial order on the 4-dimensional feature space by **x**≤**y** iff x_i_≤y_i_ for all i, and the above monotonicity assumptions then take the simple form **x**≤**y** implies P(active|**x**)≤P(active|**y**).

Finally, there is one additional subtlety that all implementations of THEMATICS have had to address, and the current approach is no exception: the need for some kind of normalization across proteins. In Ko's approach [3], the raw features were individually transformed into Z-scores, the deviations from the mean in units of the standard deviation, as calculated for the set of all ionizable residues within a given protein. Similarly, in Tong's SVM approach [5], the raw features were likewise transformed into robust Z-scores, defined as the deviations from the median in units of the interquartile distance for the set of all ionizable residues within a given protein. Here a very different type of transformation is applied to each feature across the population of residues within a given protein. We call this transformation rank normalization. Within each protein, each feature value is ranked from lowest to highest in that protein, and each data point is then assigned a number uniformly across the interval [0,1] based on the rank of that feature in that protein. The highest value for that feature is thus transformed to 1, and the lowest value is transformed to 0. Note that unlike the use of Z-scores or the robust Z-scores of Tong [5], this is a nonlinear transformation of the raw feature values. For each scalar feature x, denote its within-protein rank-normalized value as 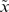, which by definition lies in [0,1]. The use of this notation is extended to feature vectors in the obvious way, *i.e.* 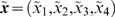.

Note that this rank normalization transformation does not affect the within-protein partial order used in the assumptions. That is, **x**≤**y** is true for raw feature vectors **x** and **y** in the same protein if and only if 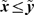. However, when data from multiple proteins is combined for training and the results are used to make predictions for new proteins, as is described in detail below, this actually implies an even stronger monotonicity assumption across proteins in which the within-protein rankings replace the raw feature values. This assumption is harder to justify intuitively, but such an approach is required to be able to train on multiple proteins and make predictions for novel proteins, and, as shown below, it appears to give good results.

### Maximum Likelihood Probability Estimation under Monotonicity Constraints: The POOL Method

After the normalization is performed, the labeled dataset may be regarded as a collection of 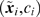 pairs, one for each ionizable residue in the protein, where 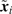 is the 4-dimensional rank-normalized feature vector for the ith residue and the label c_i_ is either 1 (identified as an active-site residue) or 0 (not so identified). Given such a set of training data, the mathematical problem we wish to solve is to find a maximum likelihood estimator for 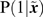 as a function of 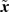 in [0,1]^4^ based on this training dataset and satisfying the constraint that 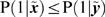 whenever 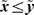 in the coordinate-wise partial order described above. Letting n represent the number of training examples and p_i_ the estimate of 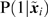 for each i from 1 to n, we seek to maximize:
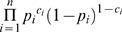
(2)subject to the constraints: 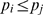 for each *(i,j)* such that 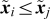


This is a convex optimization problem with linear constraints. We have shown [8] that the solution to this convex optimization problem is the same as the solution to the quadratic programming problem of the minimization of:
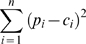
(3)subject to these same constraints. Note that while the equivalence of these solutions is well-known if there are no constraints, not every constrained maximum-likelihood problem is equivalent to the corresponding minimum squared-error problem with the same constraints. However, with these particular constraints, the two solutions are indeed identical.

This latter optimization problem is a special case of the general *isotonic regression* problem [9],[10] and this special form lends itself to a particularly straightforward solution technique. First, at an arbitrary point in the feasible region, the set of active constraints is determined by solving the corresponding dual quadratic programming problem of finding {λ_i_} minimizing
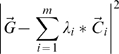
subject to the constraints λ_i_≥0 for all i, where 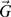 is the negative gradient and 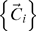 are the normal vectors to the m constraint surfaces.

The kth constraint in the primal problem is active iff λ_k_>0. Furthermore, by rescaling coordinates in the primal problem, its contours become circular and the negative gradient at any point points toward a single point, the unconstrained optimum. (Thus another formulation of the primal problem is to find the point in the feasible region closest to the unconstrained optimum.) As a consequence, the active set so determined at any feasible point is exactly the same as the active set at the solution point. But the active set simply represents equivalence classes of data points for which equality of the estimates must hold. Since equality-constrained maximum-likelihood estimates have the form (number of positives)/(total number of points), identifying which constraints are active at the solution leads immediately to the solution itself. Full details of this algorithm as well as the proof that the minimum sum-of-squared-errors solution is also the maximum-likelihood solution can be found in Tong's dissertation [8].

We call our algorithm for solving this maximum-likelihood problem the POOL algorithm. POOL is both an acronym for Partial Order Optimal Likelihood as well as an accurate characterization of the way the method first identifies the active constraints and then simply combines the corresponding data values into “pools” to be assigned probability estimates according to the proportion of positives in that pool.

The use of the POOL method with this 4-dimensional THEMATICS feature vector is denoted POOL(T4) in the Results section, where its performance is compared with other methods.

### Combining THEMATICS Data and Cleft Size Rank with POOL

Previous studies have shown that active site residues tend to be located in one of the largest clefts in a protein structure [6],[7],[11]. Indeed it has been reported that in 83% of single-chain enzymes, the active site is located in the largest cleft [11]. Nearly all active sites are principally located in one of the five largest clefts of a protein structure, with the largest cleft containing the active site for the highest fraction of enzymes and with the fractions decreasing as the size rank progresses to smaller clefts [12]. If such purely geometric analyses were to be used for active site prediction, the result would be low precision and a high false positive rate, since the active site typically constitutes just a fraction of the area of the cleft. Since such geometric analyses are purely 3D-structure based, may be performed rapidly, and constitute a very different type of information from that of THEMATICS, it makes sense to combine these data in order to enhance overall performance. One straightforward way to do this is to combine the features from both THEMATICS and cleft size rank into a single vector of input to any appropriate classifier or probability estimator. Cleft size rankings may be readily incorporated into POOL, since there is an implicit monotonicity assumption that applies to this feature as well: The probability that a cleft contains an interaction site is highest for the largest cleft in a protein structure and decreases for clefts of smaller size rank. In this study we used CASTp [13], which uses computational geometry to define and measure pockets on the protein surface, to calculate cleft information for each residue in the protein. For present purposes, every residue in a given protein is assigned an integer number corresponding to the rank of the size of the cleft to which it belongs, where 1 is the largest, 2 is the second-largest, and so on. If the atoms of a residue belong to more than one cleft, the residue is assigned the rank of the largest of these clefts. When combined with THEMATICS features, the result is a 5-dimensional input vector to which coordinate-wise monotonicity constraints are applied on all five coordinates. POOL(T4,G) denotes the estimator resulting from applying the POOL method to this five-dimensional concatenation of the four THEMATICS features and one cleft size rank.

An interesting alternative to simply concatenating all features into a single vector and applying a single classifier or probability estimator to such vectors is to compute two separate probability estimates and then combine them. Consider the general problem of estimating the class probability P(c|**x**) for a feature vector **x** = (**x**_1_, **x**_2_, …, **x**_k_) formed as the concatenation of feature vectors **x**_1_, **x**_2_, …, **x**_k_. It is straightforward to show that if the naïve Bayes conditional independence assumption
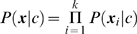
(4)holds for each class c, then
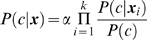
(5)where α is a normalizing constant. This gives a computationally attractive way to consider combining probability estimates for combinations of feature sets when separate estimates are available for the individual feature sets. As with other applications of naïve Bayes, it is not necessary that the conditional independence assumption be strictly true for the results of this computation to give useful results, especially when it comes to relative rankings [14].

This then gives another approach, which we have dubbed *chaining*, to obtain active-site probability estimates using both THEMATICS features and cleft size rank. In this case, we use equation (5) to combine the POOL estimates based on THEMATICS with the POOL estimates for the one-dimensional cleft size rank feature. POOL estimates based on the four-dimensional THEMATICS input and those based on the one-dimensional cleft size rank are labeled POOL(T4) and POOL(G), respectively, where G stands for geometry. POOL(G) gives a simple set of active-site probabilities for each ranking. The probability estimator computed using equation (5) with POOL(T4) and POOL(G) we then call POOL(T4)xPOOL(G). Later we also incorporate a conservation score feature, based on sequence alignment, using this same technique.

### Extension of THEMATICS to Non-Ionizable Residues with POOL

Non-ionizable residues do not have titration curves and thus THEMATICS does not predict them directly. Nevertheless, the non-ionizable residues in interaction sites tend to have ionizable residues in their immediate vicinity and these ionizable residues generally have perturbed titration curves [1],[5]. This was the basis for the attempt by Tong *et al.* [5] to identify non-ionizable active site candidate residues by their proximity to the ionizable residues selected by THEMATICS. That approach, based on SVM results and called SVM-region, yields an unacceptably high false positive rate. Here we adopt a related strategy based on POOL and demonstrate substantially improved results.

Note that every non-ionizable residue has the environment features μ_3_^env^ and μ_4_^env^; these serve as measures of the overall amount of titration curve perturbation in their spatial neighborhood. Thus we posit an extension to the THEMATICS monotonicity assumptions, namely: All other things being equal, a non-ionizable residue having more titration curve perturbation in its neighborhood is more likely to be an active-site residue. Thus we can apply the POOL method to non-ionizable residues separately by applying coordinate-wise monotonicity constraints to the probability estimates for the 2-dimensional feature vectors 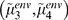, once again using the transformations, rank-normalized within each protein, of these features. In this case, the rank normalization is performed separately on just the set of non-ionizable residues in a given protein.

Furthermore, we have the same options described above for incorporating cleft or other information for these non-ionizable residues. Finally, for any given protein, we can start with separate ordered lists of probability estimates for the ionizables and the non-ionizables, however computed, and then merge these into a single ordered list. This list then gives an estimated probability, and hence a ranking, for all residues.

### Incorporating Sequence Conservation Information

Yet another feature that is generally taken to be predictive of functional activity in a monotonic fashion is the extent to which a given residue is found to be conserved across sequence homologues: The more conserved the residue, the more likely that residue is to be functionally important in the protein. Here we also examine combining such conservation information with THEMATICS and cleft size information. In particular, we use ConSurf [15], a sequence comparison based method that identifies functionally important regions on the surface of a protein of known three-dimensional structure, based on the phylogenetic relations between its close sequence homologues. If there are a sufficient number of sufficiently diverse homologues to the query protein, Consurf assigns a score between 1 and 9 to each residue in the query sequence based on how conserved this residue is among those homologues. The more conserved a residue is, the higher its score. We call this the conservation feature and in the results below we use *C* to denote its inclusion. Note that taken by itself, it is also a simple one-dimensional feature, like the cleft size rank. In this study, if Consurf returns more than 10 homologues, we use the score ConSurf assigns to each residue as its conservation feature value. For any proteins with 10 or fewer homologues, all residues in that protein are assigned a single common value for that feature. The effect of this is that all residues in such proteins have a tie for that feature, so it contributes nothing to the individual probability estimates or the residue rankings within that protein.

In the Results section it is shown that using information derived solely from the 3D structure, the present method outperforms all other 3D structure based methods. When sequence conservation information is included, the resulting classifier outperforms all other methods. Especially noteworthy is that in the absence of sequence conservation information, performance is nearly as good as that with such conservation information. This is particularly significant for structural genomics proteins, for which the present method is expected to perform well, even for novel folds and orphan sequences.

## Results

As described in more detail in the Materials and Methods section, the results presented in this paper are based on two sets of proteins, a set of 64 test proteins selected randomly from the CSA database [16],[17] and a 160-protein set covering most of the original CSA database. A detailed list of the names of the proteins, the PDB IDs of the structures, the E.C. classification, and the CSA-labeled positive residues within each protein in both test sets can be found in the Supporting Information; Dataset S1 contains the 64-protein test set and Dataset S2 contains the 160-protein test set. For each set of performance data reported here, the results are based on eight-fold cross-validation for the 64-protein set and ten-fold cross-validation for the-160 protein set.

### Performance Measures: ROC Curves

The results presented here are based on several standard measures of performance. For a standard classification problem, performance is typically measured by *recall* (or *true positive rate*) and *false-positive rate*. Within a specific system with tunable parameters, recall and false positive rate typically involve a tradeoff: adjusting the parameters to lower the false-positive rate also lowers recall, while raising the latter also raises the former. So to judge the performance of such a system, it is important to know the tradeoff between these two, and thus *ROC curves*, which plot recall against false positive rate, are presented here. In the latter subsections, it is sometimes necessary to use other performance measures in order to compare our results against those reported by others.

Since our method outputs a ranked list (actually a list of probabilities) for all residues within a given protein and not a binary classification, considering the ROC curves is an especially useful way to characterize the behavior of *any* binary classification scheme derived from it. Among the many possibilities for creating a binary classification from such a list would be to select the top *n* or the top *p* percent or use a probability threshold. One advantage of ROC curves is that they are independent of the selection scheme.

One disadvantage of using ROC curves alone, however, is that unless the curve for one method dominates (i.e., lies completely above and to the left of) that of another, there may be no simple metric to compare these two methods. For this reason, a single number that is sometimes used as a reliable measure for comparing systems in the machine learning literature is the *area under the ROC curve* (*AUC*) [18]. We make use of this as a single numerical measure to which we can apply statistical significance tests to corroborate the apparent superiority of one method over another.

In order to generate ROC curves, we need to be able to calculate recall and false-positive rate values, which come from classification problems. In the POOL method, the result for each protein is a ranked list based on the probability of a residue being in the active site. A natural way to draw a ROC curve for every protein is to move the cutoff one residue at a time from the top to the bottom of the list. The resulting ROC curve has a staircase shape: only recall increases when an active site residue is encountered and only false positive rate increases when a non-active-site residue is encountered.

We define *average specificity* (*AveS*) for each protein in the set:
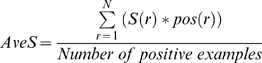
(6)where *r* is the rank, *N* is the number of residues in a protein, *pos(r)* is a binary function that indicates whether the residue of a given rank *r* is annotated in the reference database in the active site (pos(*r*) = 1) or not (pos(*r*) = 0), and *S(r)* is the *specificity* at a given cut-off rank *r*. (*Specificity* is defined to be 1 – false positive rate.) It is not hard to see that *AveS* represents the area under the ROC curve (AUC) for that protein. We also compute the across-protein mean of *AveS* over a given set of proteins, which we call the *mean average specificity* (*MAS*) for that set.

To visually compare the performance from different methods, we also generate the *averaged ROC curve* for each method by computing the recall and false-positive rate after truncating the list after each of the positive residues in turn, followed by linearly interpolating the value at each recall value and computing the mean of the interpolated false-positive rate values across all proteins in the dataset.

From these average ROC curves we can get a strong sense of the apparent relative performance of different systems, but it is also important to be able to verify that such apparent differences are in fact statistically significant. To test the significance of the observed differences, we also perform the Wilcoxon signed-rank test [19] on *AveS* from these methods to estimate the probability of observing such a difference under the null hypothesis that the seemingly better-performing method is actually not better than the other. This test essentially determines which method is consistently better on a protein-by-protein basis (as measured by AUC or *AveS*), while the curves we display essentially demonstrate which methods perform better on average.

#### Ionizable residues using only THEMATICS features

**Figure 1:**
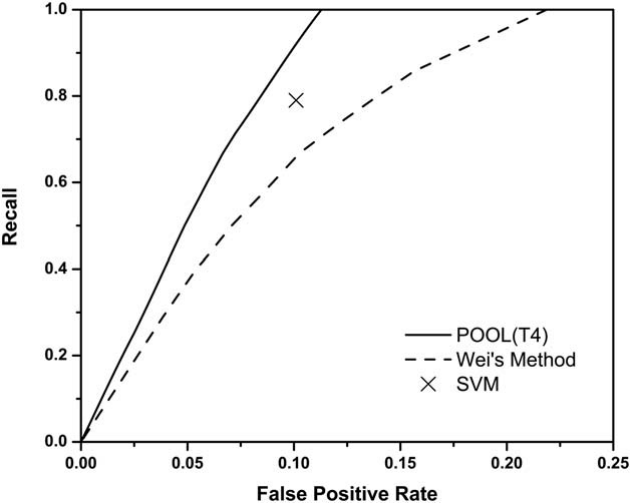
Prediction of annotated ionizable active site residues in a test set of 64 proteins using only THEMATICS features. Shown in the plot are the averaged ROC curves, recall as a function of false positive rate, for POOL(T4) (solid curve) and Wei's statistical analysis (dashed curve) along with Tong's SVM (point X). Predictions all use THEMATICS features on ionizable residues only; performance is measured using annotated active site ionizable residues. POOL(T4) outperforms both the SVM and Wei's method.

**Figure 2:**
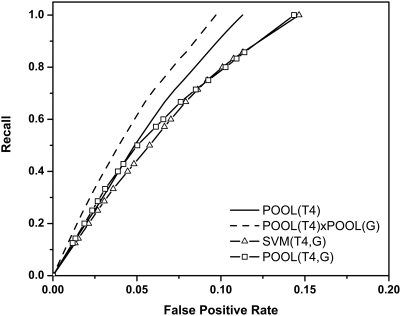
Prediction of annotated ionizable active site residues in a test set of 64 proteins using both THEMATICS and cleft information. Averaged ROC curves comparing different methods of predicting ionizable active site residues using a combination of THEMATICS and geometric features of ionizable residues only. The POOL(T4)xPOOL(G) method using chaining to combine both THEMATICS features and geometric information (dashed curve) performs better than POOL with THEMATICS features alone (solid curve), POOL on a 5D concatenated feature space (□), and an SVM on a 5D feature space (triangles).

Here we evaluate the ability of POOL with the four THEMATICS features, denoted *POOL(T4)*, to predict ionizable residues in the active site. For the purposes of Figures 1 and 2, only the *ionizable* CSA-annotated active site residues are taken as the labeled positives. Thus if a method successfully predicts all of the labeled ionizable active residues, its true positive rate is 100%. The prediction of all active residues, including the non-ionizable ones, is addressed below.

Figure 1 shows the ROC curve obtained using POOL(T4), with just the four-dimensional THEMATICS feature vectors described earlier (solid curve) for the 64-protein test set. As noted above, the POOL method computes maximum-likelihood probability estimates, but for these ROC curves only the rankings of all residues within a single protein matter. For comparison, Figure 1 also shows a corresponding ROC curve for the earlier THEMATICS-Statistical approach introduced by Ko *et al.* [3] and refined by Wei *et al.* [4] (dashed curve), plus the single point (X) corresponding to the THEMATICS-SVM approach [5]. The dataset used for the THEMATICS-Statistical curve consists of the same 64 proteins used here. Note that the POOL(T4) curve always lies above and to the left of the statistical curve for all non-zero values of recall. For any given non-zero value of the false positive rate (FPR), the recall is always higher for POOL(T4) than for the statistical method. The point representing the particular SVM classifier is based on a separate set of data, trained and tested on datasets somewhat different from the present dataset, so the results are not strictly comparable. Nevertheless, this point lies well below the POOL(T4) curve and strongly suggests that POOL(T4) is superior to the SVM approach [5]. Below we present further evidence that POOL outperforms an SVM on this active-site classification task. Thus POOL(T4) appears to represent our best method yet for identifying ionizable active-site residues using THEMATICS features alone.

#### Ionizable residues using THEMATICS plus cleft information

Next we evaluate three different ways of combining THEMATICS features with cleft size information. Figure 2 shows averaged ROC curves for these three different combinations, along with the best-performing THEMATICS-only method, POOL(T4) (solid curve), for the 64-protein test set. The three methods are: (i) POOL(T4,G), which uses the POOL method with the 5-dimensional concatenated feature vectors of THEMATICS and cleft size rank (where *G* represents the *geometric* feature); (ii) SVM(T4,G), which uses a support vector machine trained using the same 5-dimensional feature vectors, with varying threshold; and (iii) POOL(T4)xPOOL(G) (dashed curve), the result of chaining POOL(T4) estimates with POOL(G) estimates.

To compare the averaged ROC curves from Figure 2 quantitatively, we computed the area under the curve for each ROC curve in the figure using the *mean average specificity* (*MAS*). The *MAS* values for POOL(T4)xPOOL(G), POOL(T4), POOL(T4,G) and SVM(T4,G) are 0.939, 0.921, 0.909 and 0.903, respectively. Figure 2 and these *MAS* values provide a comparison of average performance between these different methods. In order to estimate the statistical significance of the performance difference considering all pairwise comparison results (*i.e.*, on a per-protein basis), we performed the Wilcoxon signed-rank test. Table 1 shows the p-value of the Wilcoxon signed-rank test, the probability of observing the specified *AveS* measurement with the null hypothesis that the method listed in the corresponding row does not out-perform the method listed in the corresponding column, as the first number in each cell. The number N in parentheses indicates the number of proteins out of the 64, for which the method in that row outperforms the method in that column. For the remaining (64-N) proteins in the set, the two methods either give equal performance or the method in the column outperforms the method in the row.

**Table 1:** Wilcoxon signed-rank tests between methods shown in Figure 2.

Method	SVM(T4,G)	POOL(T4,G)	POOL(T4)
POOL(T4)xPOOL(G)	<0.0001 (53)	<0.0001 (59)	<0.0001 (46)
POOL(T4)	0.0002 (40)	0.0006 (41)	
POOL(T4,G)	0.038 (37)		

The first number in each cell is the Wilcoxon p value, the probability that the method in the corresponding row does not outperform the method in the corresponding column. The number in parentheses is the number of proteins out of 64 for which the method in the row outperforms the method in the column.

Figure 2 and Table 1 clearly show that chaining the POOL(T4) and POOL(G) probability estimates is the method that gives the best performance. It is interesting to note that this method, POOL(T4)xPOOL(G), is the only one that outperforms POOL(T4) alone. It is also interesting to note that POOL(T4) is consistently at least as good as SVM(T4,G), and is significantly better than SVM(T4,G) in the upper recall range, even though the latter has the advantage of the additional cleft information. In general, there is little difference between POOL(T4), SVM(T4,G), and POOL(T4,G) in the lower recall range, but for recall above about 0.6, POOL(T4) has a significantly lower false positive rate, on average, than the other two, given equal recall. So these ROC curves and corresponding statistical tests provide strong evidence that POOL(T4)xPOOL(G) is the only one of the methods reported to date that is capable of taking good advantage of additional geometric information that is not contained in THEMATICS features alone and thereby outperforms any purely THEMATICS-based method so far.

The better performance of this chained method POOL(T4)xPOOL(G) over POOL(T4) alone is consistent throughout the ROC curve. For recall rates greater than 0.50, the recall for the chained method is better than that of POOL(T4) by roughly 10% for a given FPR. This qualitative trend is apparent from visual inspection of the ranked lists from the two methods. For a typical protein, these two ranked lists tend to be very similar, with annotated positive residues generally ranking a little higher, on average, in the list resulting from chaining.

We believe that the observation that chaining the two four- and one- dimensional estimators gives better results than applying POOL directly to the single, five-dimensional concatenated feature vector is probably an overfitting issue. There may be too much flexibility when POOL is used with a high-dimensional input space, especially when the data are sparse.

#### All residues using THEMATICS plus cleft information

**Figure 3:**
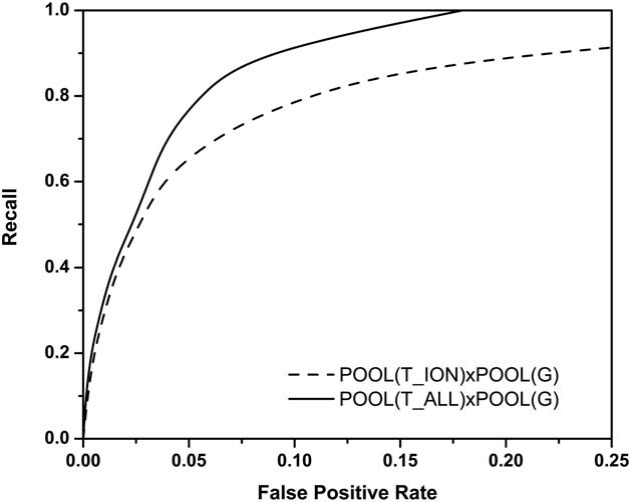
Averaged ROC curves for two versions of the POOL method, one that predicts ionizable residues only POOL(T_ION_)xPOOL(G) and the other that predicts all residues POOL(T_ALL_)xPOOL(G) through the incorporation of environment variables. Recall rate for all annotated active site residues is plotted as a function of the false positive rate for all residues in the 64 protein test set.

So far only predictions for ionizable residues have been described. The THEMATICS environment variables are now used to incorporate predictions for non-ionizable residues in the active site. Figure 3 shows the ROC curve for a combined method by which a single merged, rank-ordered list of all residues, both ionizable and non-ionizable, in a protein is generated. The method assigns probability estimates for ionizable residues using the best of the previous ionizables-only estimators, the estimator corresponding to the best ROC curve POOL(T4)xPOOL(G) in Figure 2. It also assigns probability estimates to non-ionizable residues using POOL with the two THEMATICS environment features chained with POOL(G), and then rank orders all the residues based on their probability estimates. We give this new estimator obtained by merging these ionizable-only probability estimates with these non-ionizable-only probability estimates the name *POOL(T_ALL_)xPOOL(G)*. Also included in Figure 3 for comparison is a ROC curve for POOL(T_ION_)xPOOL(G) based on the same estimates for the ionizable residues but assigning probability estimates of zero to all non-ionizable residues. Note that the data for this latter method are essentially the same as those of the POOL(T4)xPOOL(G) curve of Figure 2, except that the denominator for the recall values is now the number of total active-site residues in the protein, whether ionizable or not, and the denominator for the false positive rate is now the total number of non-active-site residues in the protein, ionizable or not. The improved ROC curve for the merged estimate method POOL(T_ALL_)xPOOL(G) compared to the curve for the ionizables-only method POOL(T_ION_)xPOOL(G) indicates that taking into account both THEMATICS environment variables and cleft information does indeed help identify the non-ionizable active-site residues. When the lists are merged, the rankings of some annotated positive ionizable residues may be lowered, but it is apparent that this effect is more than offset, on average, by the inclusion in the ranking of some annotated, positive, non-ionizable residues that are obviously missed by excluding them altogether. If this were not the case, then one would expect the merged curve to cross below (and to the right of) the comparison curve in the lower recall (and lower false positive) range.

The *MAS* values for POOL(T_ALL_)xPOOL(G) and POOL(T_ION_)xPOOL(G) are 0.933 and 0.833, respectively. The p-value of the Wilcoxon signed-rank test of observing such *AveS* under the null hypothesis that the POOL(T_ALL_)xPOOL(G) does not outperform POOL(T_ION_)xPOOL(G) is <0.0001. It is further noted that POOL(T_ALL_)xPOOL(G) outperforms POOL(T_ION_)xPOOL(G) in 31 of the 64 proteins. The number of proteins for which POOL(T_ALL_)xPOOL(G) outperforms POOL(T_ION_)xPOOL(G) in this case may seem low, but both methods perform the same in 25 out of the 64 proteins. For many of these latter cases, the protein does not have any non-ionizable residues in the active site.

This shows that this extension of the POOL method to non-ionizable residues gives a satisfactory result. From now on, all residues are included in the study and we further simplify our feature set naming convention to use *T* to indicate the way THEMATICS is used in T_ALL_: for ionizable residues, the probability estimates are obtained by using the POOL method on all four THEMATICS features; for non-ionizable residues, these probability estimates are obtained by applying the POOL method using just the two environment features.

#### All residues using THEMATICS, cleft information, and sequence conservation

So far we have only considered 3D-structure-based active-site residue prediction. This is important because such methods are applicable to cases where sequence-based methods may not apply. For many structural genomics proteins, the number of homologues is too small to obtain meaningful sequence-based conservation information. Nevertheless, since it is generally true that most active site residues tend to be more conserved than other residues, it is obviously valuable to be able to include sequence conservation information when it is available. Here we examine to what extent adding sequence comparison information can improve active-site residue prediction within the POOL framework.

**Figure 4:**
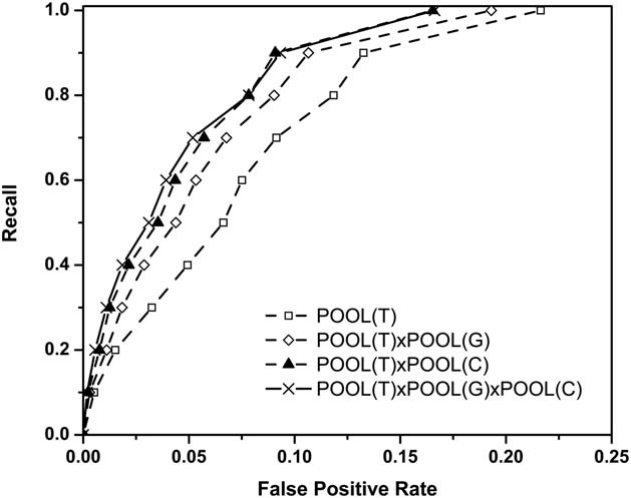
Averaged ROC curves comparing different methods of combining POOL input features: THEMATICS, geometric, and sequence conservation data, for all residues in a test set of 160 proteins. The method using chaining to combine THEMATICS, geometric and sequence conservation features has the best performance.

Figure 4 shows the ROC curves using different feature combinations on the 160-protein set, with all residues (not just ionizables) included. Here T, representing input to POOL, stands for the four THEMATICS features for ionizable residues and the two THEMATICS environment features for the non-ionizable residues; POOL(T)xPOOL(G) uses both the THEMATICS and geometric (cleft) features; POOL(T)xPOOL(C) uses both THEMATICS and the sequence conservation information; while POOL(T)xPOOL(G)xPOOL(C) uses all three features. Figure 2 has already suggested that chaining lower-dimensional POOL estimators gives better results than the application of POOL directly to concatenated feature vectors of higher dimension and therefore the chained combinations are shown for all of these cases that utilize different types of input data.

As pointed out earlier, not all proteins have enough homologues to perform reliable sequence conservation analysis. In this study, ConSurf was used to do the sequence analysis. However we only used this ConSurf result for proteins having more than 10 homologues. For those not having enough homologues (28 out of 160 proteins in the test set), a common nonzero value was assigned as the active-site probability estimate based on that feature alone. This has the same effect as ignoring this feature for these cases.

Figure 4 shows, among all four curves, that POOL(T) is dominated by all three other curves, suggesting that including either cleft or sequence conservation features, or both, gives better performance than THEMATICS features alone. Both of the curves that include conservation, POOL(T)xPOOL(C) and POOL(T)xPOOL(G)xPOOL(C), dominate POOL(T)xPOOL(G), suggesting that incorporating sequence conservation information does improve performance more than just incorporating cleft information alone. Surprisingly, POOL(T)xPOOL(C) and POOL(T)xPOOL(G)xPOOL(C) have very similar performance, although in the recall range below 80%, POOL(T)xPOOL(G)xPOOL(C) shows slightly better performance.

The *MAS* for POOL(T)xPOOL(G)xPOOL(C), POOL(T)xPOOL(C), POOL(T)xPOOL(G), and POOL(T) are 0.925, 0.923, 0.907 and 0.899, respectively. The p-values of the Wilcoxon signed-rank test of observing such *AveS* measurement with null hypothesis that the method in the row does not outperform the method in the column are listed in Table 2, as the first number in each cell. The number in the parentheses indicates the number of proteins out of 160 for which the method in that row outperforms the method in that column.

**Table 2:** Wilcoxon signed-rank tests between methods shown in Figure 4.

Method	POOL(T)	POOL(T)xPOOL(G)	POOL(T)xPOOL(C)
POOL(T)xPOOL(G)xPOOL(C)	<0.0001 (115)	<0.0001 (95)	<0.0001 (103)
POOL(T)xPOOL(C)	<0.0001 (101)	0.0008 (89)	
POOL(T)xPOOL(G)	<0.0001 (101)		

Numbers in parentheses give the actual number of proteins out of 160 for which the method in that row outperforms the method in that column in the AUC measure.

#### Recall-filtration ratio curves

The results reported so far are all in the form of *ROC curves*. As discussed earlier, this analysis is not committed to any particular cutoff or rule to select the active site residues from the top of the list. For instance, users can select the top *k* residues in the ranked list of residues ordered by the estimated probability of being in the active site, or they can select the residues with an estimated probability of being in the active site greater than a certain cutoff value, or they can select the top *p* percent of the residues in the ranked list. Among the three cutoff criteria listed above, we focus here on the third approach, partly because we need to commit to some way of creating a binary classifier to do the comparisons with some other methods from the literature for which the data for ROC curves has not been provided.

**Figure 5:**
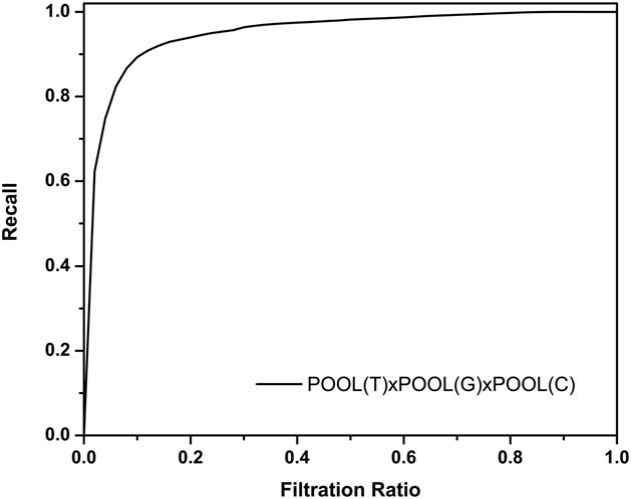
Averaged recall as a function of Filtration Ratio (RFR) curve for POOL(T)xPOOL(G)xPOOL(C) for all residues in the 160 protein test set.

Note that neither axis of a ROC curve involves a directly user-controllable parameter. Neither recall nor false positive rate is under the direct control of a user who does not already know the correct classifications. Assuming the user wishes to select the highest-ranking values in the list, down to a certain fixed proportion, a more useful curve would be a *recall-filtration ratio (RFR)* curve, where filtration ratio is defined to be the fraction of all residues predicted as positive. Figure 5 shows an averaged RFR curve for the best-performing POOL(T)xPOOL(G)xPOOL(C) method for the 160-protein test set. In this case, the vertical axis is the average recall (across proteins) obtained when the proportion of predicted positives is set at the value on the horizontal axis. For the curve shown in Figure 5, for example, choosing the top 10% of the residues from the ranked list gives an average recall of 90%, while choosing the top 5% of the residues from the ranked list gives an average recall of 79%.

#### Comparison with other methods

Here results are compared for our best structure-only method, POOL(T)xPOOL(G), and for our best structure-plus-sequence method, POOL(T)xPOOL(G)xPOOL(C), with the results from some other top performing active site prediction methods, particularly, Petrova's method [20], Youn's method [21], and Xie's geometric potential method [22]. The first two use both sequence conservation and 3D structural information, while Xie's method uses 3D structural information only. Petrova's method and Youn's method are both based on Support Vector Machines.

The authors of the three methods report their performance results using a variety of different measures, often different from what we have reported here. Therefore we simply compute corresponding results, using their form of analysis, for POOL(T)xPOOL(G) and POOL(T)xPOOL(G)xPOOL(C) on our 160 protein test set and compare our numbers with theirs. Because the performance measures are not achieved from the same dataset, results are not strictly comparable, but qualitatively, we believe the comparisons below give a good idea of the relative performance.

In order to compare our results with theirs at a similar recall level, we used a 4% filtration ratio cutoff in the POOL method to compare with Youn's method, and a variable filtration ratio cutoff to compare with Petrova's method. Note that while our test set consists of proteins with a wide variety of different folds and functions, Youn's results are reported for sets of proteins with common fold or with similar structure and function. Performance on the more varied set is a much more realistic test of predictive capability on proteins of unknown function, particularly novel folds. Performance on a set of structurally or functionally related proteins is also substantially better than performance on a diverse set, as one would expect and as has been demonstrated by Petrova and Wu [20].

Youn's method [21] achieved about 57% recall at 18.5% precision with *MAS* (*AUC*) of 0.929, using both sequence conservation and structural information when they train and test on proteins from the same family; however the performance dropped when the training and testing is performed on proteins of the same superfamily and fold level, while our POOL(T)xPOOL(G)xPOOL(C) with a preset 4% filtration ratio cutoff, achieves the averaged recall of 64.68% with averaged precision of 19.07%, and an *MAS* (*AUC*) of 0.925 for all 160 proteins in the test set, consisting of proteins from completely different folds and classes. Without the use of sequence conservation, POOL(T)xPOOL(G) achieves averaged recall, averaged precision and *AUC* of 61.74%, 18.06% and 0.907, respectively. Without conservation information, our chained POOL method achieves recall and precision rates that are at least as good as those of Youn's method, even though the latter does include conservation information. POOL with conservation information included obtains better recall and precision than Youn's reported values, even though our diverse test set is one for which good performance is most difficult to achieve. The complete results are shown in Table 3.

**Table 3:** Comparison of sensitivity, precision, and AUC of the chained combination POOL(T)xPOOL(G)xPOOL(C) on our diverse test set of proteins with Youn's reported results for proteins in the same family, superfamily, and fold.

Method/Dataset	Sensitivity (%)	Precision (%)	AUC
Youn/Family	57.02	18.51	0.9290
Youn/Superfamily	53.93	16.90	0.9135
Youn/Fold	51.11	17.13	0.9144
POOL(T)xPOOL(G)xPOOL(C)/all protein	64.68	19.07	0.925
POOL(T)xPOOL(G)/all protein	61.74	18.06	0.907

**Figure 6:**
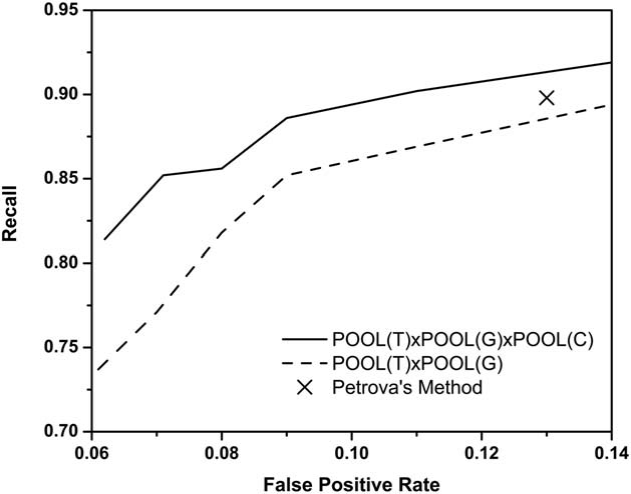
ROC curves comparing POOL(T)xPOOL(G), POOL(T)xPOOL(G)xPOOL(C), and Petrova's method (X). POOL results are for a 72 protein test set.

Petrova and Wu [20] measured the performance of their method globally using all residues in all proteins, instead of computing the recall, accuracy, false positive rate and Matthews correlation coefficient (MCC) values for each protein and then averaging them. Like Youn's method, they use both sequence conservation information and 3D structural properties as input to the SVM. They use a dataset that they call the benchmarking dataset that contains a wide variety of proteins that are dissimilar in sequence, are structurally diverse, and span the full range of E.C. classes of chemical functions. This dataset constitutes a fair test of how a method will perform on structural genomics proteins of unknown function for which sequence conservation information is available. Their method achieves a global residue level 89.8% recall with an overall predictive accuracy of 86%, with an MCC of 0.23 and a 13% false positive rate on a subset of 79 proteins from the CatRes database. Testing on the 72 proteins from their set that also appear in our 160 protein set, POOL(T)xPOOL(G)xPOOL(C) with a 10% filtration ratio cutoff achieves a residue level 88.6% recall at the overall predictive accuracy of 91.0%, with an MCC of 0.28 and a 9% false positive rate. Without any conservation information, POOL does about as well as Petrova and Wu: POOL(T)xPOOL(G) with a 10% filtration ratio cutoff gives a residue level recall, overall predictive accuracy, MCC, and false positive rate of 85.2%, 91.0%, 0.27, and 9%, respectively. When conservation information is added to POOL, the results improve a little. The ROC curves in Figure 6 show recall and false positive rates for POOL as POOL(T)xPOOL(G) (dashed curve) and POOL(T)xPOOL(G)xPOOL(C) (solid curve); the reported performance of the method of Petrova and Wu on a very similar set of annotated proteins is shown as an X on the ROC curve.

POOL with 3D structure input information only, employed as POOL(T)xPOOL(G), predicts active site residues without any sequence alignment information and performs nearly as well as the very best methods to date that do use sequence alignment information.

In the method of Xie and Bourne [22], a purely 3D structure based method, the performance was reported in the following fashion: their method achieves at least a 50% recall with 20% or less false positive rate for 85% of the proteins they analyzed. The performance of the POOL(T)xPOOL(G) and POOL(T)xPOOL(G)xPOOL(C) methods measured in the same way is listed in Table 4. Xie's method should be compared against POOL(T)xPOOL(G), because these methods do not use conservation data. POOL(T)xPOOL(G) achieves at least a 50% recall with a false positive rate of 20% or less for 96% of all proteins.

**Table 4:** Comparison of POOL(T)xPOOL(G) and POOL(T)xPOOL(G)xPOOL(C) with Xie's method.

Method	Recall ≥	False Positive Rate <	Achieved For
Xie	50%	20%	85%
POOL(T)xPOOL(G)xPOOL(C)	50%	20%	97%
POOL(T)xPOOL(G)xPOOL(C)	80%	20%	84%
POOL(T)xPOOL(G)xPOOL(C)	60%	10%	85%
POOL(T)xPOOL(G)	50%	20%	96%
POOL(T)xPOOL(G)	80%	20%	77%
POOL(T)xPOOL(G)	60%	10%	81%

Each method achieves at least the specified recall rate with a false positive rate less than specified for the percentage of proteins in the last column.

The results in the tables clearly show that POOL(T)xPOOL(G), which only uses 3D structural information of proteins, achieves about as good or even better performance than that of these best performing current active site prediction methods. When additional sequence conservation information is available, still better performance is achieveable with POOL(T)xPOOL(G)xPOOL(C).

#### Rank of the first positive

**Figure 7:**
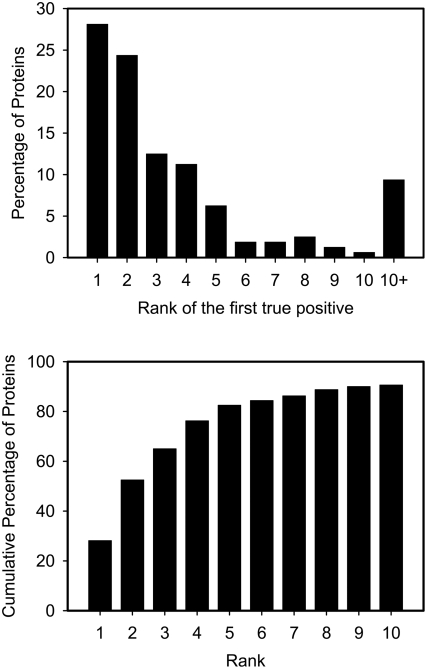
Histogram of the first annotated active site residue. Top: Percentage of all proteins with specified rank of the first annotated active site residue in the ordered list from POOL(T)xPOOL(G)xPOOL(C) on the 160 protein set. Bottom: Cumulative distribution of the first annotated active site residue in the ranked list from POOL(T)xPOOL(G)xPOOL(C) on the 160 protein set.

Another interesting result of our approach is one that is only obtainable from methods that generate a ranked list: the rank of the first annotated true positive in the list. This metric is useful for users who are interested in finding a few of the active site residue candidates and who do not necessarily need to know all of the active site residues. For instance, users could use the list from the POOL method to guide their site directed mutagenesis experiments by going down the ranked list one by one. A histogram giving the rank of the first active site residue found by POOL(T)xPOOL(G)xPOOL(C) on the 160 protein set is shown in Figure 7. The median rank of the first true positive active site residue in the 160 protein set with POOL(T)xPOOL(G)xPOOL(C) method is two. For 46 out of 160 proteins, the first residue in the resulting ranked list is an annotated active site residue. 65.0%, 81.3% and 90.0% of the 160 proteins have the first annotated active site residue located within the top 3, 5 and 10 residues of the ranked list, respectively. Such measurements are not easily made for binary classification methods.

#### Cases where annotated residues rank low

To identify the proteins for which POOL performs poorly, we shall set a filtration ratio cutoff of 8.0% for this purpose and use the CSA-annotated residues as the reference. The top 8% of POOL-ranked residues contain one or more CSA-annotated residues for 156 (97.5%) of the 160 proteins in the test set. It is useful to examine the four other cases where the CSA-annotated active residues rank low. These are considered failure cases and consist of: Phenol hydroxylase from *Trichosporon cutaneum* (PDB ID 1FOH); bovine Acylphosphatase (PDB ID 2ACY); Adenine-N6-DNA-methyltransferase from *Thermus aquaticus* (PDB ID 2ADM); and Serine carboxypeptidase II from wheat (PDB ID 1BCR).

Phenol hydroxylase (ΨOH) uses the cofactor flavin adenine dinucleotide (FAD) to hydroxylate phenols [23]. The crystal structure contains phenol and FAD. The three CSA annotated residues, D54, R281, and Y289, are all ranked low by POOL. However the phenol-binding residues P364 and K365 have high POOL rankings, as do the FAD binding residues V13, G14, G16, C224, D225, S229, Y336, and G369. Thus POOL does select a number of residues in the site of interaction, although it is unable to find the CSA annotated residues. ΨOH is one unusual instance where the optimized statistical THEMATICS selector of Wei [4] performs better than POOL relative to the CSA annotations, as Wei's method successfully identifies D54.

The structure of Acylphosphatase contains only 98 residues and two sulfate ions; presumably the sulfate ions indicate phosphate binding sites. Neither POOL nor the statistical version of THEMATICS is able to identify the two CSA annotated residues R23 and N41. However Wei's statistical method does correctly identify two sulfate-contact residues, K32 and H60. These two residues both have low POOL rankings. Adenine-N6-DNA-methyltransferase and Serine carboxypeptidase II both have a relatively large number of residues involved in binding and recognition; POOL returns low rankings for the annotated residues. We note that POOL does well for other cases with relatively large numbers of residues involved in the site of interaction. Indeed at this time no pattern is discernable that distinguishes the small set of failure cases from the large group of successful cases for POOL.

#### Relative contributions of the different features

**Figure 8:**
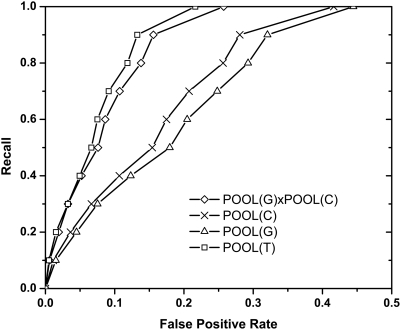
Averaged ROC curves for POOL(G), POOL(C), and POOL(G)xPOOL(C), shown with that for POOL(T) for comparison (which is also shown in Figure 4) for a 160 protein test set. This demonstrates the relative contributions made to the combined system by these different features. Clearly there is a domination order: THEMATICS alone, then the combination of conservation and geometric information, then conservation alone, then geometric alone.

Looking at Figure 2, one can note that of the averaged ROC curves displayed there, there are three feature combinations not represented. These are the feature combinations that do not include the THEMATICS features, namely POOL(C), POOL(G), and POOL(G)xPOOL(C). That is, nowhere in our analysis up to this point have we considered the application of the POOL method using only conservation information, only geometric information, or a combination of these two. Figure 8 shows the averaged ROC curves for these three feature combinations not including THEMATICS. The averaged ROC curve for POOL(T), shown earlier in Figure 4, is also shown again here for comparison. Visually, one can see that there is an apparent domination order POOL(T), POOL(G)xPOOL(C), POOL(C), and then POOL(G). The Wilcoxon test validates this apparent order with at a high level of statistical significance (<10^−4^). Note that these results can be combined with those displayed in Figure 4 to give a transitive domination order for all seven non-empty combinations of these three sets of features.

This bears out some widely recognized observations: that cleft size information is useful, but suffers from an inordinately large false positive rate and that conservation information is more useful [24]. It also shows that combining conservation information with cleft information gives a better result than either alone, but it is interesting that THEMATICS alone does better than this combination. Thus it appears that in the high-performance method we have presented here combining all three types of information, THEMATICS features represent the single most important contribution to active-site residue prediction, followed by sequence conservation features and then cleft size information.

## Discussion

In this paper, we presented the application of the POOL method using THEMATICS plus some other features for protein active site prediction.

We started with the application of the POOL method just on THEMATICS features, with features similar to those used before in the SVM method [5], as well as those used in Ko and Wei's statistical analysis [3],[4]. These results show that the POOL method outperforms all of the earlier THEMATICS methods with no cleaning of the training data and no clustering after the classification. This suggests that by relying solely on the underlying THEMATICS monotonicity assumptions, the POOL method makes better use of the training data.

We also tested different ways of incorporating additional features into the learning system. Not surprisingly, the results show that in order to improve performance, we have to incorporate the right features in the right way. Even with features that were found to be helpful in improving the performance, how they are incorporated matters. The data show that chaining the results from separate POOL estimates is better than simply combining all the available features into a big POOL estimator over a higher-dimensional feature space. As mentioned earlier, the reason behind this might be overfitting, since combining features into a POOL table with high dimension causes the number of probabilities needed for estimation to grow exponentially, while the training data can only increase linearly in most cases. In other words, the high dimensionality makes the table too sparse and less accurate for probability estimates.

We also extended the application of THEMATICS to all residues, not just ionizable residues, in a natural way and showed that it is effective. Although the performance for non-ionizable residues is not as good as the performance for ionizable ones, this extension does provide a way to combine features from THEMATICS, which by itself can only be applied to ionizable residues directly, with some other features. The inclusion of the non-ionizable residues results in better overall performance and also makes performance comparison with other methods more accurate and fair.

The incorporation of sequence conservation information does improve the predictions when there are enough homologues with appropriate diversity. The POOL method gives us a means for easily utilizing this information when it is available, while not affecting the training and classification when it is not.

When comparing with other methods, especially if the other methods use binary classification instead of a ranked list, we have to commit to a specific cutoff value and turn our system into a binary classification system. The results in this paper clearly show that the POOL method using THEMATICS and geometric features achieves equivalent or better performance than the other methods in comparison, even in cases where their methods are tested on very special groups of proteins. This makes this method more widely applicable to proteins with few or no sequence homologues, such as some Structural Genomics proteins, than other methods that use sequence alignments from homologues. Performances of the previous best methods, those of Youn and of Petrova, will degrade significantly when sequence conservation information is not available. However with THEMATICS data the approach developed here is still robust in the absence of sequence conservation information. In effect, for those proteins having an insufficient number of sequence homologues, the POOL(T)xPOOL(G)xPOOL(C) method reduces to the still highly effective structure-only POOL(T)xPOOL(G) method.

Interestingly enough, when comparing the performance of POOL(T)xPOOL(G) and POOL(T)xPOOL(G)xPOOL(C) in Figure 4, it is apparent that the addition of the conservation information does improve the performance a little, but not to the extent observed previously for sequence-structure methods. Typically the conservation information is the most important input feature, and without it performance is substantially worse [24]. This suggests that the 3D structure based THEMATICS features are quite powerful compared with other 3D structure based features and can take the place of conservation information for purposes of active site prediction. This is also borne out by the analysis at the end of the Results section.

When looking at the recall and false positive rates of the results from all the protein active site prediction methods, one must keep in mind that the annotation of the catalytic residues in the protein dataset is never perfect. Since most of the labeling comes from experimental evidence, some active site residues are not labeled as positive simply because no experiment was ever carried out to verify the role of that specific residue. Since we have used the CatRes/CSA annotations as the sole criteria to evaluate the performance in order to keep the comparisons consistent, the reported false positive rate is probably higher than in reality. There is evidence available to support the functional importance of some residues that are not labeled as active in the CatRes/CSA database [3],[4] and these residues have high ranks in the list from the POOL method and are classified as positive by THEMATICS-SVM and THEMATICS-statistical analysis as well.

Although we evaluated the POOL method performance using filtration ratio values as a cutoff, it is just for the purpose of comparing with other protein active site prediction methods that use a binary classification scheme. The ranked list of residues based on their probability of being in the active site contains much more information than traditional binary classification. The rank of the first annotated positive residue analysis in this paper shows just one application of the extra information contained in a ranked list rather than a traditional binary label. There are many possible measurements of performance depending on the actual application by users, and in turn many possible applications that can benefit from using a ranked list form. It is noteworthy that P-Cats [25] also estimates the probability that a residue belongs to a protein active site, using a k-nearest neighbor method. The P-Cats server uses the probability estimates as the basis to assign binary labels; residues with probability larger than 0.50 are labeled as positive and the others as negative. The method of Cheng [26] also generates a rank-ordered list based on a scoring system; these scores could in principle be translated into probability estimates.

The POOL approach is amenable to the addition of other properties for the prediction of active sites [27]–[32]. We also note that the POOL methodology is applicable to other types of problems in a variety of different areas where probability depends monotonically on the input feature variables.

In conclusion, we have established that applying the POOL method, with THEMATICS and other features, appears to yield the best protein active site prediction system yet found and it provides more information than other active site prediction methods.

## Materials and Methods

The three-dimensional coordinate files for the protein structures used for training and testing were downloaded from the Protein Data Bank (http://www.rcsb.org/pdb/). In order to predict the theoretical titration curve of each ionizable residue in the structure, finite-difference Poisson-Boltzmann calculations were performed using UHBD [33] on each protein followed by the program HYBRID [34], which calculates a corresponding titration curve of the form average net charge as a function of pH. These titration curves were obtained for each ionizable residue: Arg, Asp, Cys, Glu, His, Lys, Tyr, and the N- and C- termini. The pH range we simulated for all curves is from −15.0 to 30.0, in increments of 0.2 pH units. This wide theoretical pH range is necessary for proper numerical integration of the first derivative functions. The structures were processed and analyzed to obtain the central moments μ_3_ and μ_4_, as described earlier. These individual features were then rank-normalized within each protein, and thus assigned values in the interval [0,1], also as described above. This four-dimensional representation constitutes what we designate the *THEMATICS features* for each residue. The monotonicity assumptions for this multidimensional feature set are as described earlier.

For the *geometric feature*, we used CASTp [13], which uses a pocket algorithm for shape measurements to calculate the cleft information for each residue in the protein. The clefts were ranked based on their sizes in decreasing order and each residue having atoms located in any cleft is assigned the rank number of the largest of the clefts where its atoms are located. One special value is assigned to every residue not on the protein surface, and another is assigned to every residue on the surface but not within any cleft. Ignoring these special values, the monotonicity assumption is that the larger the cleft to which a residue belongs, the more likely that residue is to belong to the active site.

For the *conservation feature* we used ConSurf [15] to calculate a sequence conservation score for the residues in each protein. ConSurf takes a protein sequence and finds its closest sequence homologues using MUSCLE [35], a multiple-sequence alignment algorithm. Two sequences with similarity higher than a preset threshold are treated as homologues. ConSurf analyzes the homologues of the query sequence and determines how conserved each residue is in the query protein among these homologues. In order to normalize the result and make it comparable between different proteins with different numbers of homologues and with different degrees of overall conservation, the program labels each residue with a conservation score between 1 and 9, with 9 being the most conserved and 1 being the most variable. If there exist more than 50 homologues for the query sequence, the 50 homologues closest to the query sequence are analyzed. In this study, we only used the conservation score reported by ConSurf when there are at least 11 homologues for a protein. The monotonicity assumption applied to this feature is that the larger the conservation score for a residue, the more likely that residue is to belong to the active site.

The results reported here are based on eight-fold cross-validation on a set of 64 proteins or 10-fold cross-validation on a set of 160 proteins, both taken from the Catalytic Site Atlas (CSA) database [16],[17]. The labels were taken directly from the CSA database; if a residue is identified there as active in catalysis, it was labeled as positive in our dataset. If not so identified in the CSA, we labeled it as negative. The CSA annotations, although incomplete, constitute the best source of active residue labels for enzymes. In anticipation that the POOL method would not be overly sensitive to mislabeled data, no hand tuning of the labels was performed and no residues were omitted during training, in contrast to the SVM study reported by Tong [5].

For the eight-fold cross-validation procedure, the 64-protein set was randomly divided into eight folds of eight proteins each, with seven of the eight folds (56 proteins) used for training and the remaining fold (8 proteins) used for testing. This was repeated eight times, once for each of the eight folds. Likewise, for the ten-fold cross-validation procedure, the 160-protein set was randomly divided into ten folds of sixteen proteins each, with nine of these (144 proteins) used for training the remaining fold (16 proteins) used for testing, and this was repeated a total of ten times, once for each fold.

Training was performed by applying the POOL method to obtain a function 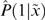 for each rank-normalized feature vector 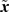 in the appropriate feature space [0,1]^k^. Note that: k = 4 for the POOL method applied on the four THEMATICS features of ionizable residues as stated earlier, denoted by POOL(T4); k = 5 for the POOL method applied on the four THEMATICS features of ionizable residues plus the geometric feature of the cleft size, denoted as POOL(T4,G); k = 1 for the POOL method applied to the geometric feature of cleft size, denoted by POOL(G), as well as the POOL method applied to the conservation feature, denoted by POOL(C); and k = 2 for POOL applied to the environmental features for non-ionizable residues, denoted as POOL(T2).

An additional detail is that for training we quantize the multi-dimensional data points. For example, for POOL(T4), each rank-normalized feature falls into one of 20 bins whose sizes vary depending on their distance from 0.0. In particular, the lowest ranked bins cover the half-open intervals, [0.0, 0. 2), [0. 2, 0.4), [0.4, 0.6), [0.6, 0.7), and there are 16 more bins of width 0.02 above that, with one special bin for 1.0. Thus the lowest-ranking data are quantized more coarsely than the remaining data. This is appropriate since these data tend to have very low average probability of being in the active site anyway, because the vast majority of residues are negatives. Thus the inability to make fine distinctions among these low-probability candidates does not degrade the overall quality of the results. It does, however, improve the efficiency of the training procedure significantly, so this is an important component of the analysis. This is especially helpful in the 10-fold cross-validation on the 160-protein set. The typical training set of 144 proteins from this set contains about 14500 ionizable residues, which fall into more than 6000 quantized bins in the 4-dimensional space used for POOL(T4). The number of corresponding inequality constraints is about 35,000–40,000.

One final detail is that the probability estimates generated by the POOL method as described here tend to have numerous ties as well as some places where there is no well-defined value. The latter places occur because the method only assigns values to existing data points (or bins containing data in the case of our use of quantization). The locally constant regions occur both because of the quantization applied to the training data at the outset and because the data pools created by the algorithm acquire a single value. In cells where no value is defined, the interpolation scheme used is to simply assign a value linearly interpolated based on the Manhattan distance between the least upper bound and the greatest lower bound for that cell based on the monotonicity constraint. Finally, since both the data pooling performed by the algorithm and this interpolation scheme tend to lead to ties, the Manhattan distance from the origin of the four THEMATICS features is used as a tie-breaker for any residues whose probability estimates are identical. This simply imposes a slight bias toward strict monotonicity even though the mathematical formulation used to determine these probabilities is based on a non-strict monotonicity assumption, making it possible to obtain well-defined rankings for all the residues in a protein.

## Supporting Information

Dataset S1 The 64 protein test set used for POOL
(0.08 MB DOC)

Dataset S2 The 160 protein test set used for POOL
(0.18 MB DOC)
