# Supplementary material for: Partial Order Optimum Likelihood (POOL): Maximum Likelihood Prediction of Protein Active Site Residues Using 3D Structure and Sequence Properties
Source: PLoS Comput Biol. 2009 Jan 16;5(1):e1000266. doi: 10.1371/journal.pcbi.1000266 (PMC2612599; doi:10.1371/journal.pcbi.1000266)
Supplement: Dataset S1 — The 64 protein test set used for POOL (0.08 MB DOC) [file pcbi.1000266.s001.doc]

**Dataset S1. The 64 protein test set used for POOL**

| PDB Code | Protein Name | E.C. Number | CSA Annotated Active Site Residues |
| --- | --- | --- | --- |
| 1A05 | 1,4-Diacid decarboxylating dehydrogenase | 1.1.1.85 | Y140, K190, D222 |
| 1A26 | ADP-ribosyltransferase | 2.4.2.30 | Y907, E988 |
| 1A4I | Methylenetetrahydrofolate Dehydrogenase | 1.5.1.5 | K56 |
| 1A4S | Aldehyde dehydrogenase (NAD+) / Betaine-aldehyde dehydrogenase | 1.2.1.8 | N166, E263, C297 |
| 1AFW | Acetyl-CoA C-acyltransferase | 2.3.1.16 | C125, H375, C403, G405 |
| 1AKM | Ornithine Carbamoyltransferase | 2.1.3.3 | R106, H133, Q136, D231, C273, R319 |
| 1AOP | Sulphite reductase | 1.8.1.2 | R83, R153, K215, K217 C483 |
| 1APX | Heme peroxidase | 1.11.1.11 | R38, H42, N71 |
| 1B6B | Aralkylamine N-acetyltransferase | 2.3.1.87 | S97, L111, H122, L124, Y168 |
| 1BG0 | Arginine Kinase | 2.7.3.3 | R126, E225, R229, R280, R309 |
| 1BRM | Aspartate-beta-semialdehyde dehydrogenase | 1.2.1.11 | C135, Q162, H274 |
| 1BRW | Pyrimidine-nucleoside phosphorylase | 2.4.2.2 | H82, R168, S183, K187 |
| 1BWD | L-arginine:Inosamine-phosphate amidinotransferase | 2.1.4.2 | D108, R127, D179, H227, D229 H331, C332 |
| 1BZY | Hypoxanthine-guanine phosphoribosyltransferase | 2.4.2.8 | E133, D134, D137, K165, R169 |
| 1C3J | DNA beta-glucosyltransferase | 2.4.1.27 | E22, D100 |
| 1COY | Cholesterol Oxidase | 1.1.3.6 | E361, H447, N485 |
| 1CQQ | Picornain 3C | 3.4.22.28 | H40, E71, G145, C147 |
| 1D0S | Nicotinate-nucleotide-dimethylbenzimidazole phosphoribosyltransferase | 2.4.2.21 | E317 |
| 1D4A | NAD(P)H dehydrogenase (quinone) | 1.6.99.2 | G149, Y155, H161 |
| 1D4C | Succinate dehydrogenase (Fumerate reductase) | 1.3.99.1 | H364, R401, H503, R544 |
| 1DII | 4-cresol dehydrogenase | 1.17.99.1 | Y73, Y95, E380, E427, H436, R474 |
| 1DLI | UDP-glucose 6-dehydrogenase | 1.1.1.22 | T118, E145, K204, N208, C260, D264 |
| 1DO8 | Malate dehydrogenase | 1.1.1.39 | Y112, K183, D278 |
| 1E2A | Histidine Kinase IIAlac | 2.7.1.69 | H78, Q80, D81, H82 |
| 1EBF | Homoserine dehydrogenase | 1.1.1.3 | D219, K223 |
| 1FOH | Phenol 2-monooxygenase | 1.14.13.7 | D54, R281, Y289 |
| 1FUG | Methionine adenosyltransferase | 2.5.1.6 | H14, K165, R244, K245, K265, K269, D271 |
| 1G72 | Methanol dehydrogenase | 1.1.99.8 | D297 |
| 1GET | Glutathione reductase | 1.6.4.2 | C42, C47, K50, Y177, E181, H439, E444 |
| 1GOG | Galactose Oxidase | 1.1.3.9 | C228, Y272, W290, Y495 |
| 1GPR | The IIAglc Histidine kinase | 2.7.1.69 | T66, H68, H83, G85 |
| 1GRC | Phosphoribosylglycinamide formyltransferase (GARTFase II) | 2.1.2.2 | N106, H108, S135, D144 |
| 1IVH | Isovaleryl-CoA dehydrogenase | 1.3.99.10 | E254 |
| 1JDW | Glycine amidinotransferase | 2.1.4.1 | D254, H303, C407 |
| 1KAS | 3-oxoacyl-[acyl-carrier protein] synthase | 2.3.1.41 | C163, H303, H340, F400 |
| 1L9F | Monomeric sarcosine oxidase | 1.5.3.1 | H45, R49, H269, C315 |
| 1LCB | Thymidylate synthase | 2.1.1.45 | E60, R178, C198, S219, D221, D257, H259 |
| 1LXA | UDP-N-acetylglucosamine acyltransferase | 2.3.1.129 | H125 |
| 1MBB | UDP-N-acetylmuramate dehydrogenase | 1.1.1.158 | R159, S229, E325 |
| 1MHL | Mammalian Myeloperoxidase | 1.11.1.7 | Q91, H95, R239 |
| 1MLA | [Acyl-carrier protein]  S-malonyltransferase | 2.3.1.39 | S92, H201, Q250 |
| 1MOQ | Glucosamine--fructose-6-phosphate aminotransferase (isomerising domain) | 2.6.1.16 | E481, K485, E488, H504, K603 |
| 1MPY | Extradiol Catecholic Dioxygenase | 1.13.11.2 | H199, H246, Y255 |
| 1NID | Nitrite Reductase | 1.7.99.3 | D98, H255 |
| 1NSP | Nucleoside-diphosphate kinase | 2.7.4.6 | K16, N119, H122 |
| 1OFG | Glucose-fructose oxidoreductase | 1.1.99.28 | K129, Y217 |
| 1PFK | Phosphofructokinase | 2.7.1.11 | G11, R72, T125, D127, R171 |
| 1PJB | Alanine dehydrogenase | 1.4.1.1 | K74, H95, E117, D269 |
| 1PKN | Pyruvate Kinase | 2.7.1.40 | R72, R119, K269, T327, S361, E363 |
| 1PUD | Queuine tRNA-ribosyltransferase (tRNA-guanine transglycosylase) | 2.4.2.29 | D102 |
| 1R51 | Urate Oxidase | 1.7.3.3 | R176, Q228 |
| 1RA2 | Dihydrofolate reductase | 1.5.1.3 | I5, M20, D27, L28, F31, L54, I94 |
| 1UAE | UDP-N-acetylglucosamine  1-carboxyvinyltransferase | 2.5.1.7 | N23, C115, D305, R397 |
| 1ULA | Purine-nucleoside phosphorylase (type 1) | 2.4.2.1 | H86, E89, N243 |
| 1VAO | Vanillyl Alcohol Oxidase | 1.1.3.13 | Y108, D170, H422, Y503, R504 |
| 1VNC | Chloride peroxidase | 1.11.1.10 | K353, H404 |
| 1XVA | Glycine N-methyltransferase | 2.1.1.20 | E15 |
| 1ZIO | Adenylate kinase | 2.7.4.3 | K13, R127, R160, D162, D163, R171 |
| 2ALR | Mammalian Aldehyde Reductase | 1.1.1.2 | Y49, K79 |
| 2BBK | Methylamine dehydrogenase | 1.4.99.3 | D32, W57, D76, W108, Y119, T122 |
| 2CPO | Heme Chloroperoxidase | 1.11.1.10 | H105, E183 |
| 2HDH | 3-hydroxyacyl-CoA dehydrogenase | 1.1.1.35 | S137, H158, E170, N208 |
| 2JCW | Superoxide dismutase | 1.15.1.1 | H63, R143 |
| 3PCA | Protocatechuate dioxygenase | 1.13.11.3 | Y447, R457 |
