# Supplementary material for: Partial Order Optimum Likelihood (POOL): Maximum Likelihood Prediction of Protein Active Site Residues Using 3D Structure and Sequence Properties
Source: PLoS Comput Biol. 2009 Jan 16;5(1):e1000266. doi: 10.1371/journal.pcbi.1000266 (PMC2612599; doi:10.1371/journal.pcbi.1000266)
Supplement: Dataset S2 — The 160 protein test set used for POOL (0.18 MB DOC) [file pcbi.1000266.s002.doc]

**Dataset S2. The 160 protein test set used for POOL**

| PDB Code | Protein Name | E.C. Number | CSA Annotated Active Site Residues |
| --- | --- | --- | --- |
| 12AS | Aspartate--ammonia ligase | 6.3.1.1 | D46, R100, Q116 |
| 13PK | Phosphoglycerate kinase | 2.7.2.3 | R39, K219, G376, G399 |
| 1A05 | 1,4-Diacid decarboxylating dehydrogenase | 1.1.1.85 | Y140, K190, D222 |
| 1A26 | ADP-ribosyltransferase | 2.4.2.30 | Y907, E988 |
| 1A4I | Methylenetetrahydrofolate Dehydrogenase | 1.5.1.5 | K56 |
| 1A4S | Aldehyde dehydrogenase (NAD+) / Betaine-aldehyde dehydrogenase | 1.2.1.8 | N166, E263, C297 |
| 1AE7 | Phospholipase A2 (PLA2) | 3.1.1.4 | G30, H48, D99 |
| 1AFW | Acetyl-CoA C-acyltransferase | 2.3.1.16 | C125, H375, C403, G405 |
| 1AH7 | Phospholipase C | 3.1.4.3 | D55 |
| 1AKM | Ornithine Carbamoyltransferase | 2.1.3.3 | R106, H133, Q136, D231, C273, R319 |
| 1ALK | Alkaline Phosphatase | 3.1.3.1 | S102, R166 |
| 1AOP | Sulphite reductase | 1.8.1.2 | R83, R153, K215, K217 C483 |
| 1APX | Heme peroxidase | 1.11.1.11 | R38, H42, N71 |
| 1APY | Aspartylglucosylaminidase | 3.5.1.26 | T183, T201, T234, G235 |
| 1AQ2 | Phosphoenol pyruvate carboxykinase | 4.1.1.49 | H232, K254, R333 |
| 1AW8 | Aspartate 1-decarboxylase | 4.1.1.11 | Y58 |
| 1B3R | Adenosylhomocysteinase | 3.3.1.1 | D130, K185, D189, N190, C194 |
| 1B57 | Fructose-bisphosphate aldolase (class II) | 4.1.2.13 | D109, E182, N286 |
| 1B66 | 6-pyruvoyl tetrahydropterin synthase | 4.6.1.10 | C42, D88, H89, E133 |
| 1B6B | Aralkylamine N-acetyltransferase | 2.3.1.87 | S97, L111, H122, L124, Y168 |
| 1B73 | Glutamate racemase | 5.1.1.3 | D7, S8, C70, E147, C178, H180 |
| 1B93 | Methylglyoxyl synthase | 4.2.99.11 | H19, G66, D71, D91, H98, D101 |
| 1BCR | Carboxypeptidase D | 3.4.16.6 | G53, S146, Y147, D338, H397 |
| 1BG0 | Arginine Kinase | 2.7.3.3 | R126, E225, R229, R280, R309 |
| 1BJP | 4-oxalocrotonate tautomerase | 5.3.2.0 | P1, R39, F50 |
| 1BML | Plasmin/streptokinase | 3.4.21.7 | H603, S608, D646 |
| 1BOL | Ribonuclease T2 | 3.1.27.1 | H46, E105, H109 |
| 1BRM | Aspartate-beta-semialdehyde dehydrogenase | 1.2.1.11 | C135, Q162, H274 |
| 1BRW | Pyrimidine-nucleoside phosphorylase | 2.4.2.2 | H82, R168, S183, K187 |
| 1BS4 | Peptide Deformylase | 3.5.1.31 | G45, Q50, L91, E133 |
| 1BTL | Beta-Lactamase Class A | 3.5.2.6 | S70, K73, S130, E166 |
| 1BWD | L-arginine:Inosamine-phosphate amidinotransferase | 2.1.4.2 | D108, R127, D179, H227, D229 H331, C332 |
| 1BWP | 2-acetyl-1-alkylglycerophosphocholine esterase | 3.1.1.47 | S47, G74, N104, D192, H195 |
| 1BZY | Hypoxanthine-guanine phosphoribosyltransferase | 2.4.2.8 | E133, D134, D137, K165, R169 |
| 1C3C | Adenylosuccinate lyase | 4.3.2.2 | H68, H141, E275 |
| 1C3J | DNA beta-glucosyltransferase | 2.4.1.27 | E22, D100 |
| 1CB8 | Chondroitin AC lyase | 4.2.2.5 | H225, Y234, R288 |
| 1CD5 | Glucosamine-6-phosphate isomerase | 5.3.1.10 | D72, D141, H143, E148 |
| 1CHD | Protein-glutamate methylesterase | 3.1.1.61 | S164, T165, H190, M283, D286 |
| 1CHK | Chitosanase | 3.2.1.132 | E22, D40 |
| 1CHM | Creatinase | 3.5.3.3 | H232, E262, E358 |
| 1COY | Cholesterol Oxidase | 1.1.3.6 | E361, H447, N485 |
| 1CQQ | Picornain 3C | 3.4.22.28 | H40, E71, G145, C147 |
| 1CTT | Cytidine Deaminase | 3.5.4.5 | E104 |
| 1D0S | Nicotinate-nucleotide-dimethylbenzimidazole phosphoribosyltransferase | 2.4.2.21 | E317 |
| 1D4A | NAD(P)H dehydrogenase (quinone) | 1.6.99.2 | G149, Y155, H161 |
| 1D4C | Succinate dehydrogenase (Fumerate reductase) | 1.3.99.1 | H364, R401, H503, R544 |
| 1D8C | Malate synthase | 4.1.3.2 | D270, E272, R338, D631 |
| 1D8H | Polynucleotide 5'-phosphatase | 3.1.3.33 | R393, E433, K456, R458 |
| 1DAA | Aminotransferase class-IV | 2.6.1.21 | K145, E177, L201 |
| 1DAE | Dethiobiotin synthase | 6.3.3.3 | T11, K15, K37, S41 |
| 1DB3 | GDP-mannose 4,6-dehydratase | 4.2.1.47 | T132, E134, Y156, K160 |
| 1DBT | Orotidine-5'-monophosphate decarboxylase | 4.1.1.23 | D60, K62 |
| 1DCO | 4a-hydroxytetrahydrobiopterin dehydratase | 4.2.1.96 | H62, H63, H80, D89 |
| 1DGS | NAD+ dependent DNA ligase | 6.5.1.2 | K116, D118, R196, K312 |
| 1DII | 4-cresol dehydrogenase | 1.17.99.1 | Y73, Y95, E380, E427, H436, R474 |
| 1DIZ | DNA-3-methyl adenine glycosylase II | 3.2.2.21 | Y222, W272, D238 |
| 1DL2 | Mannosyl-oligosaccharide  1,2-alpha-mannosidase | 3.2.1.113 | E132, R136, D275, E435 |
| 1DLI | UDP-glucose  6-dehydrogenase | 1.1.1.22 | T118, E145, K204, N208, C260, D264 |
| 1DNK | Deoxyribonuclease I | 3.1.21.1 | E78, H134, D212, H252 |
| 1DO8 | Malate dehydrogenase | 1.1.1.39 | Y112, K183, D278 |
| 1DQS | 3-dehydroquinate synthase | 4.6.1.3 | H275 |
| 1DZR | dTDP-4-dehydrorhamnose 3,5-epimerase | 5.1.3.13 | H63, D170 |
| 1E2A | Histidine Kinase IIAlac | 2.7.1.69 | H78, Q80, D81, H82 |
| 1EBF | Homoserine dehydrogenase | 1.1.1.3 | D219, K223 |
| 1EF8 | Methylmalonyl-CoA decarboxylase | 4.1.1.41 | H66, G110, Y140 |
| 1EUG | Uridine Nucleosidase (Uracil DNA glycosylase) | 3.2.2.3 | D64, H187 |
| 1EYI | Fructose-1,6-bisphosphatase | 3.1.3.11 | D68, D74, E98 |
| 1FGH | Aconitase | 4.2.1.3 | D100, H101, H147, D165, H167, E262, H642 |
| 1FOH | Phenol 2-monooxygenase | 1.14.13.7 | D54, R281, Y289 |
| 1FRO | Lactoylglutathione lyase | 4.4.1.5 | E172 |
| 1FUA | L-fuculose-phosphate aldolase | 4.1.2.17 | E73 |
| 1FUG | Methionine adenosyltransferase | 2.5.1.6 | H14, K165, R244, K245, K265, K269, D271 |
| 1FUI | Arabinose isomerase | 5.3.1.3 | E337, D361 |
| 1G72 | Methanol dehydrogenase | 1.1.99.8 | D297 |
| 1GET | Glutathione reductase | 1.6.4.2 | C42, C47, K50, Y177, E181, H439, E444 |
| 1GIM | Adenylosuccinate synthetase | 6.3.4.4 | D13, H41, Q224 |
| 1GOG | Galactose Oxidase | 1.1.3.9 | C228, Y272, W290, Y495 |
| 1GPM | GMP synthase | 6.3.5.2 | G59, C86, Y87, H181, E183, D239 |
| 1GPR | The IIAglc Histidine kinase | 2.7.1.69 | T66, H68, H83, G85 |
| 1GRC | Phosphoribosylglycinamide formyltransferase (GARTFase II) | 2.1.2.2 | N106, H108, S135, D144 |
| 1GTP | GTP Cyclohydrolase | 3.5.4.16 | H112, H179 |
| 1HFS | Stromelysin-1 (hydrolase) | 3.4.24.17 | E202, M219 |
| 1HXQ | UDP-glucose--hexose-1-phosphate uridylyltransferase | 2.7.7.12 | C160, H164, H166, Q168 |
| 1I7D | DNA topoisomerase III | 5.99.1.2 | E7, K8, F328, R330 |
| 1IVH | Isovaleryl-CoA dehydrogenase | 1.3.99.10 | E254 |
| 1JDW | Glycine amidinotransferase | 2.1.4.1 | D254, H303, C407 |
| 1KAS | 3-oxoacyl-[acyl-carrier protein] synthase | 2.3.1.41 | C163, H303, H340, F400 |
| 1KFU | m-Calpain Form II | 3.4.22.17 | Q99, C105, H262, N286 |
| 1KRA | Urease | 3.5.1.5 | H219, D221, H320, R336 |
| 1L9F | Monomeric sarcosine oxidase | 1.5.3.1 | H45, R49, H269, C315 |
| 1LBA | T7 Lysomsome | 3.5.1.28 | Y48, K128 |
| 1LCB | Thymidylate synthase | 2.1.1.45 | E60, R178, C198, S219, D221, D257, H259 |
| 1LXA | UDP-N-acetylglucosamine acyltransferase | 2.3.1.129 | H125 |
| 1MAS | Purine nucleosidase | 3.2.2.1 | D14, N168, H241 |
| 1MBB | UDP-N-acetylmuramate dehydrogenase | 1.1.1.158 | R159, S229, E325 |
| 1MHL | Mammalian Myeloperoxidase | 1.11.1.7 | Q91, H95, R239 |
| 1MHY | Methane Monooxygenase | 1.14.13.25 | C151, T213 |
| 1MKA | 3-hydroxydecanoyl-[acyl-carrier protein] dehydratase | 4.2.1.60 | H70, V76, G79, C80, D84 |
| 1MLA | [Acyl-carrier protein]  S-malonyltransferase | 2.3.1.39 | S92, H201, Q250 |
| 1MOQ | Glucosamine--fructose-6-phosphate aminotransferase (isomerising domain) | 2.6.1.16 | E481, K485, E488, H504, K603 |
| 1MPP | Mucoropepsin | 3.4.23.23 | D32, S35, Y75, D215 |
| 1MPY | Extradiol Catecholic Dioxygenase | 1.13.11.2 | H199, H246, Y255 |
| 1NBA | Carbamoylsarcosine Amidohydrolase | 3.5.1.59 | D51, K144, A172, T173, C177 |
| 1NID | Nitrite Reductase | 1.7.99.3 | D98, H255 |
| 1NSP | Nucleoside-diphosphate kinase | 2.7.4.6 | K16, N119, H122 |
| 1NZY | Chlorobenzoate Dehalogenase | 3.8.1.6 | F64, H90, G114, W137, D145 |
| 1OFG | Glucose-fructose oxidoreductase | 1.1.99.28 | K129, Y217 |
| 1PFK | Phosphofructokinase | 2.7.1.11 | G11, R72, T125, D127, R171 |
| 1PGS | Peptide Aspartylglucosaminidase | 3.5.1.52 | D60, E206 |
| 1PJB | Alanine dehydrogenase | 1.4.1.1 | K74, H95, E117, D269 |
| 1PKN | Pyruvate Kinase | 2.7.1.40 | R72, R119, K269, T327, S361, E363 |
| 1PS1 | Pentalenene Synthase | 4.6.1.5 | F77, R157, R173, N219, K226, R230, S305, H309 |
| 1PUD | Queuine tRNA-ribosyltransferase (tRNA-guanine transglycosylase) | 2.4.2.29 | D102 |
| 1PYA | Histidine decarboxylase | 4.1.1.22 | Y62, S81, F195, E197 |
| 1PYM | Phosphoenolpyruvate mutase | 5.4.2.9 | G47, L48, D58, K120 |
| 1QFE | 3-dehydroquinate dehydratase | 4.2.1.10 | E86, H143, K170 |
| 1QPR | Quinolinate phosphoribosyltransferase (decarboxylating) (Type II) | 2.4.2.19 | R105, K140, E201, D222 |
| 1QQ5 | 2-haloacid dehalogenase | 3.8.1.2 | D8, T12, R39, N115, K147, S171, N173, F175, D176 |
| 1QUM | Deoxyribonuclease IV | 3.1.21.2 | E261 |
| 1R51 | Urate Oxidase | 1.7.3.3 | R176, Q228 |
| 1RA2 | Dihydrofolate reductase | 1.5.1.3 | I5, M20, D27, L28, F31, L54, I94 |
| 1RBL | Ribulose bisphosphate carboxylase | 4.1.1.39 | K175, K177, K201, D203, H294, H327 |
| 1REQ | Methylmalonyl-CoA mutase | 5.4.99.2 | Y89, H244, K604, D608, H610 |
| 1RPT | High molecular weight Acid Phosphatase | 3.1.3.2 | R11, H12, R15, R79, H257, D258 |
| 1SMN | Serratia marcescens nuclease | 3.1.30.2 | R87, H89, N119 |
| 1TYF | CLP Protease (clpP) | 3.4.21.92 | G68, S97, M98, H122, D171 |
| 1UAE | UDP-N-acetylglucosamine  1-carboxyvinyltransferase | 2.5.1.7 | N23, C115, D305, R397 |
| 1UAG | UDP-N-acetylmuramoylalanine--D-glutamate ligase | 6.3.2.9 | K115, N138, H183 |
| 1ULA | Purine-nucleoside phosphorylase (type 1) | 2.4.2.1 | H86, E89, N243 |
| 1UOK | Oligo-1,6-glucosidase | 3.2.1.10 | D199, E255, D329 |
| 1VAO | Vanillyl Alcohol Oxidase | 1.1.3.13 | Y108, D170, H422, Y503, R504 |
| 1VNC | Chloride peroxidase | 1.11.1.10 | K353, H404 |
| 1WGI | Inorganic pyrophosphatase | 3.6.1.1 | D117 |
| 1XVA | Glycine N-methyltransferase | 2.1.1.20 | E15 |
| 1YTW | Protein Tyrosine Phosphatase | 3.1.3.48 | E290, D356, H402, C403, R409, T410 |
| 1ZIO | Adenylate kinase | 2.7.4.3 | K13, R127, R160, D162, D163, R171 |
| 2ACY | Acylphosphatase | 3.6.1.7 | R23, N41 |
| 2ADM | ADENINE-N6-DNA-METHYLTRANSFERASE | 2.1.1.72 | N105, P106, Y108 |
| 2ALR | Mammalian Aldehyde Reductase | 1.1.1.2 | Y49, K79 |
| 2BBK | Methylamine dehydrogenase | 1.4.99.3 | D32, W57, D76, W108, Y119, T122 |
| 2BMI | METALLO-BETA-LACTAMASE | 3.5.2.6 | D86, N176 |
| 2CPO | Heme Chloroperoxidase | 1.11.1.10 | H105, E183 |
| 2HDH | 3-hydroxyacyl-CoA dehydrogenase | 1.1.1.35 | S137, H158, E170, N208 |
| 2HGS | Glutathione Synthase | 6.3.2.3 | R125, S151, G369, R450 |
| 2JCW | Superoxide dismutase | 1.15.1.1 | H63, R143 |
| 2PDA | Pyruvate synthase | 1.2.7.1 | E64 |
| 2PFL | Formate C-acetyltransferase | 2.3.1.54 | W333, C418, C419, G734 |
| 2PHK | Protein Serine/threonine kinase | 2.7.1.38 | D149, K151 |
| 2PLC | 1-phosphatidylinositol phosphodiesterase | 3.1.4.10 | H45, D46, R84, H93, D278 |
| 2THI | Thiamine pyridinylase | 2.5.1.2 | C113, E241 |
| 3CSM | Chorismate Mutase | 5.4.99.5 | R16, R157, K168, E246 |
| 3ECA | Asparaginase/Glutaminase | 3.5.1.1 | T12, Y25, T89, D90, K162 |
| 3PCA | Protocatechuate dioxygenase | 1.13.11.3 | Y447, R457 |
| 4KBP | Purple Acid Phosphatase | 3.1.3.2 | H202, H295, H296 |
| 5COX | Prostaglandin-Endoperoxide Synthase | 1.14.99.1 | Q203, H207, Y385 |
| 5ENL | Enolase | 4.2.1.11 | E168, E211, K345, H373 |
| 5FIT | diadenosine P1, P3-triphosphate (ApppA) hydrolase | 3.6.1.29 | Q83, H94, H96 |
| 8TLN | Metalloproteinase M4 | 3.4.24.27 | E143, H231 |
| 9PAP | Thiol-Endopeptidase | 3.4.22.2 | Q19, C25, H159, N175 |
